# Supplementary material for: Heterovalent-doping-enabled atom-displacement fluctuation leads to ultrahigh energy-storage density in AgNbO3-based multilayer capacitors
Source: Nat Commun. 2023 Mar 1;14:1166. doi: 10.1038/s41467-023-36919-w (PMC9978025; doi:10.1038/s41467-023-36919-w)
Supplement: Supplementary file 1 — Supplementary Information [file 41467_2023_36919_MOESM1_ESM.pdf]

Heterovalent-doping-enabled atom-displacement fluctuation leads to ultrahigh energy-storage density in $AgNbO_3$-based multilayer capacitors

Li-Feng Zhu[1], Shiqing Deng[2]*, Lei Zhao[3], Gen Li[4], Qi Wang[1], Linhai Li[1], Yongke Yan[5]*, He Qi[2], Bo-Ping Zhang[1]*, Jun Chen[2], Jing-Feng Li[4]*

[1]School of Materials Science and Engineering, University of Science and Technology Beijing, Beijing 100083, China.

[2] Beijing Advanced Innovation Center for Materials Genome Engineering, University of Science and Technology Beijing, Beijing 100083, China.

[3] College Physics Science & Technology, Hebei University, Baoding 071002, China.

[4] State Key Laboratory of New Ceramics and Fine Processing, School of Materials Science and Engineering, Tsinghua University, Beijing, 100084, China.

[5] Electronic Materials Research Laboratory, Key Laboratory of the Ministry of Education & International Center for Dielectric Research, School of Electronic Science and Engineering, Xi'an Jiaotong University, Xi'an 710049, P.R. China

Corresponding author: sqdeng@ustb.edu.cn (S.Q. Deng); bpzhang@ustb.edu.cn (B.P. Zhang); yanyongke@xjtu.edu.cn (Y. Yan) jingfeng@mail.tsinghua.edu.cn (J.F. Li)

Table of Contents

1. Fig.S1 Schematic diagram of cationic periodic variation for ANT AFE system, and its large electric-field-induced strain caused by phase transition between AFE structure and FE one. (Page 3)
2. Fig.S2 Schematic diagram of MLCCs, and finite-element simulations for the strain distribution of AN-based, ANT-based and SANT-based MLCCs at different external electric field (*E*). (Page 4)
3. Fig.S3 HAADF-STEM image of ANT capacitors. (Page5)
4. Fig.S4 SEM image of ANT and SANT MLCCs. (Page 6)
5. Fig.S5 XRD of AN, ANT and SANT ceramics. (Page 6)
6. Unipolar P-E loop for AN MLCCs (Fig.S6), ANT MLCCs (Fig.S7) and SANT MLCCs (Fig.S8) measured at different electric fields. (Page 7-8)
7. References (Page 9)

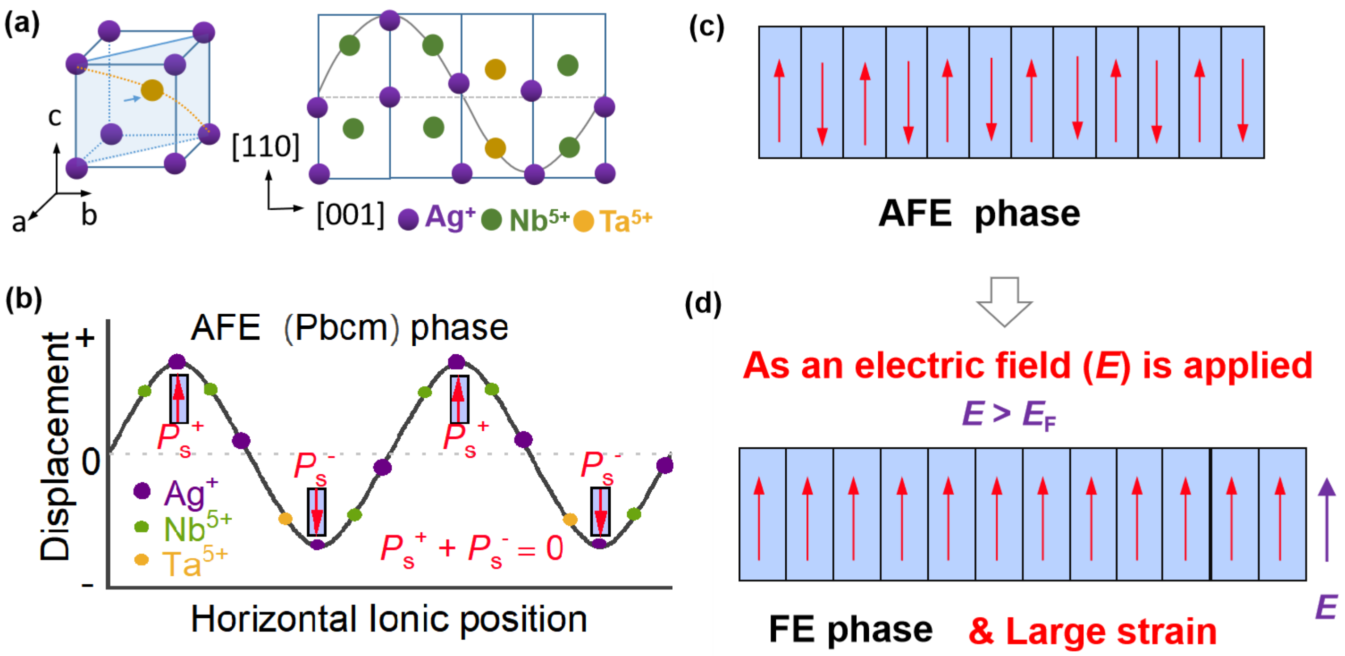

Fig.S1 Diagram of large electric-field-induced strain production for ANT system. crystal structure for ANT (a) in which the Nb^{5+} ions are replaced by Ta^{5+} ions, and two-dimensional planar plan for cationic periodic variation along the c axis direction, corresponding to the antiferroelectric Pbcm phase with cations showing displacements along the $\pm[1\bar{1}0]_C$ direction, forming a periodic variation along the c-axis direction (b). Because of the replacement Nb^{5+} by Ta^{5+} ions, the phase structure of $Ag(Nb_{0.7}Ta_{0.3})O_3$ capacitors has turned into an AFE phase, in which both its P_S^+ and P_S^- are equal and their direction is opposite [1-4]. Diagram of the volume expansion process for ANT system caused by phase transition from AFE to FE phase before (c) and after (d) applying electric field $E > E_F$. When an electric field (E, $E > E_F$) is applied in the $Ag(Nb_{0.7}Ta_{0.3})O_3$ AFE capacitors, a phase transition from AFE to FE happens, along with a large volume expansion and electric-field-induced strain[5-7].

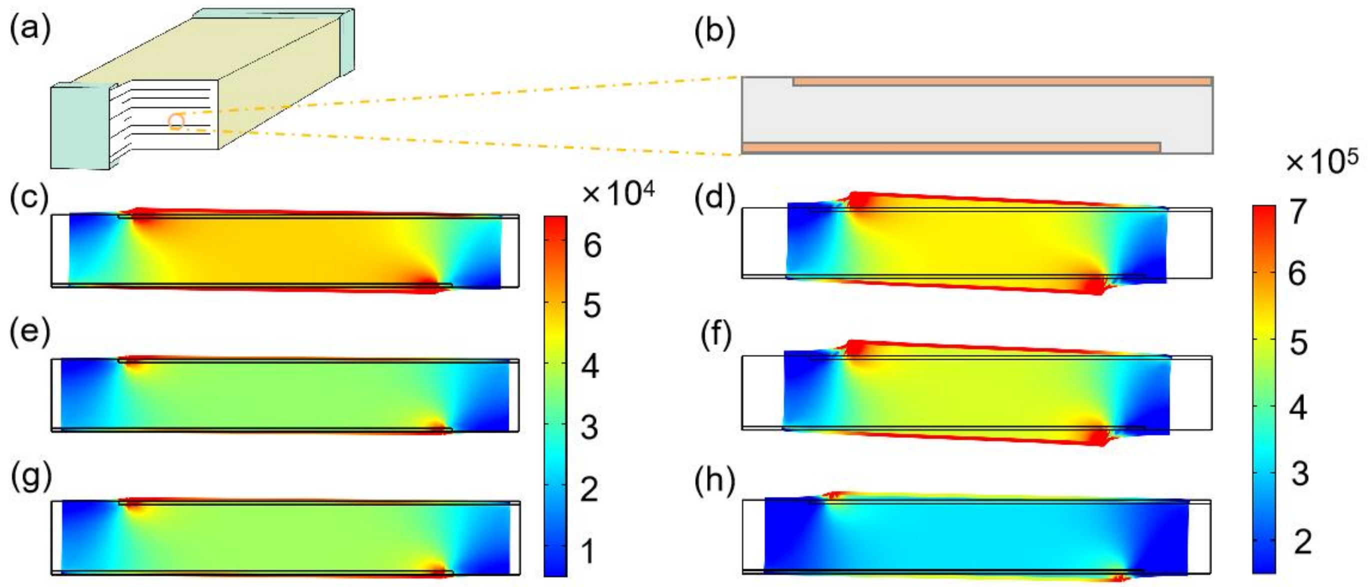

Fig.S2 Finite-element simulations for the strain distribution of MLCCs. $AgNbO_3$ MLCCs diagram (a), single layer of MLCCs (b), in which the orange and gray areas belong to Pt electrode and $AgNbO_3$ dielectric ceramics, whose thicknesses are 2 and 10 μm, respectively. Finite-element simulations for the strain distribution of AN-based, ANT-based and SANT-based MLCCs at different external electric field (E). von Miss stress distribution for AN MLCCs (N/m^2) at $E < E_F$ (c) and $E > E_F$ (d), ANT-based MLCCs $E < E_F$ (e) and $E > E_F$ (f), SANT-based MLCCs at $E < E_F$ (g) and $E > E_F$ (h), respectively.

Table S1 the parameter of finite-element simulations for AN-based, ANT and SANT MLCCs

		Flexibility matrix						Relative dielectric constant		
Materials	E	S_{11}	S_{12}	S_{13}	S_{33}	S_{44}	S_{66}	ε_{11}	ε_{22}	ε_{33}
AN	$E < E_F$	1.17	-0.335	-0.537	1.52	3.04	2.97	320	320	305
	$E > E_F$	1.25	-0.358	-0.573	1.62	3.25	3.17	430	430	415
ANT	$E < E_F$	1.18	-0.338	-0.532	1.51	3.02	2.94	430	430	410
	$E > E_F$	1.24	-0.355	-0.568	1.61	3.22	3.14	405	405	380
SANT	$E < E_F$	1.20	-0.344	-0.550	1.56	3.12	3.04	445	445	420
	$E > E_F$	1.23	-0.353	-0.564	1.59	3.20	3.12	420	420	405

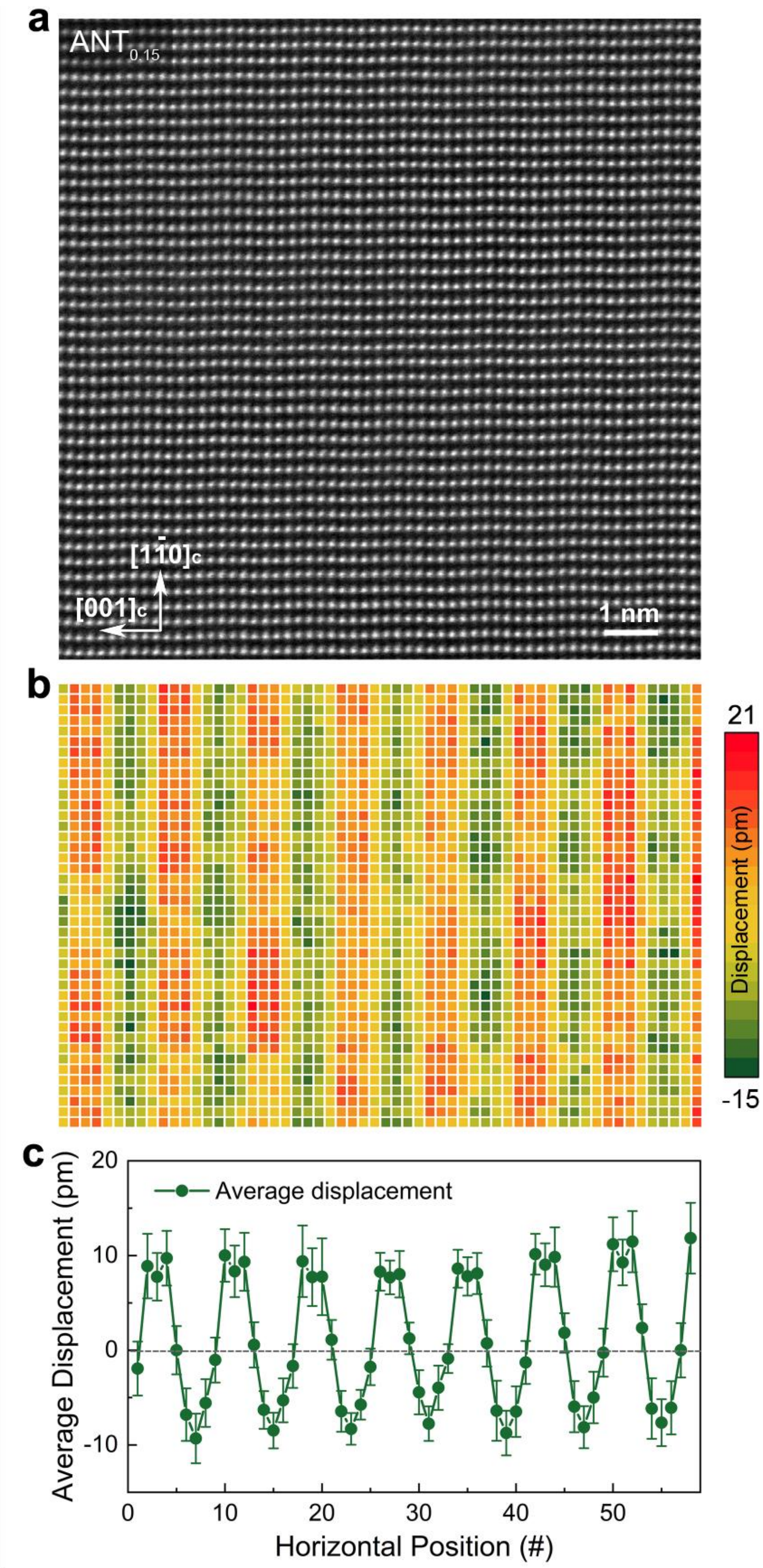

Fig.S3 Atomic-scale microstructures of ANT. HAADF-STEM image of $Ag(Nb_{0.85}Ta_{0.15})O_3$ ($ANT_{0.15}$) (a), (b) Map of the atomic displacements of Ag, Nb and Ta atoms along the [1-10]c direction, showing a good c-axial periodicity. (c) Average displacement of each vertical atomic plane. From these Figures, it can find that the LAD region is absent in the ANT system.

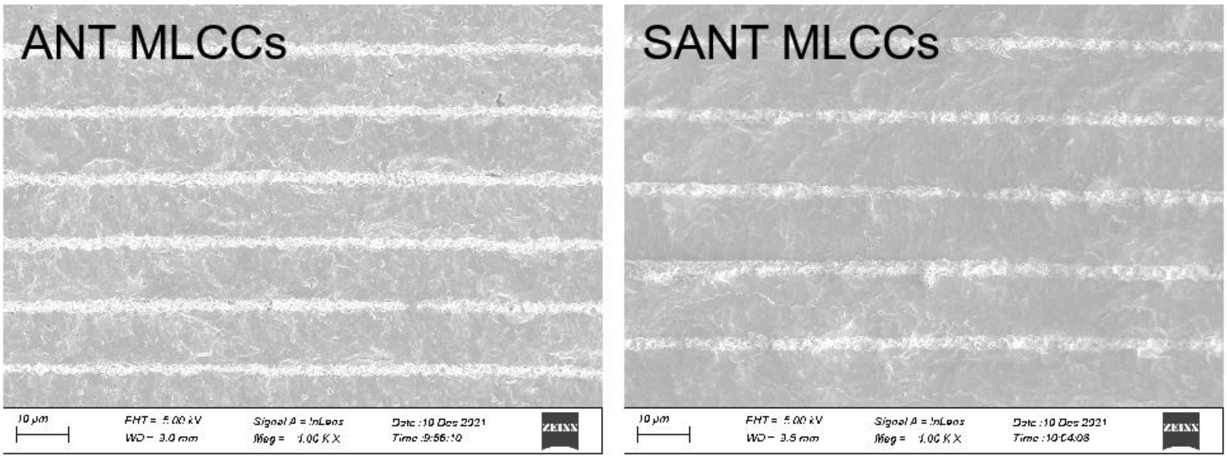

Fig.S4 SEM image of ANT and SANT MLCCs. All samples present a high density and homogeneous grain size which is less than 5 μm. The thicknesses of every dielectric layer and Pt electrode are about 9.5 μm and 2μm, respectively, in ANT MLCCs. However, the thicknesses of every dielectric layer and Pt electrode are about 10 μm and 2μm, respectively.

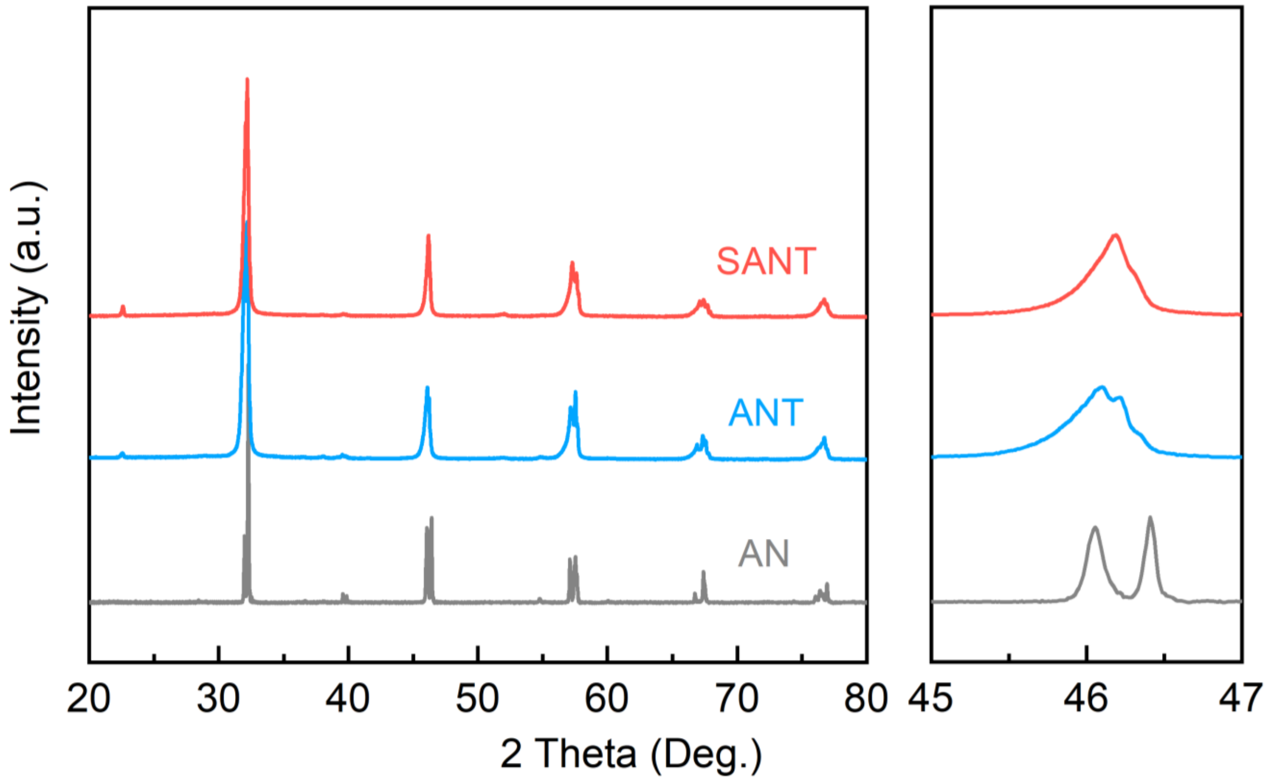

Fig.S5 XRD of AN, ANT and SANT ceramics. All samples exhibit a pure perovskite structure without any trace of impurity phase within the detectable limit of the XRD, suggesting the formation of a stable solid solution. The diffraction peaks of AN ceramic correspond well to that of M1 symmetry, in which two peaks are observed around 46°. However, for ANT and SANT ceramics, only one broad peak around 46° was detected, suggesting their phase structures have changed and turned into M3 phase.

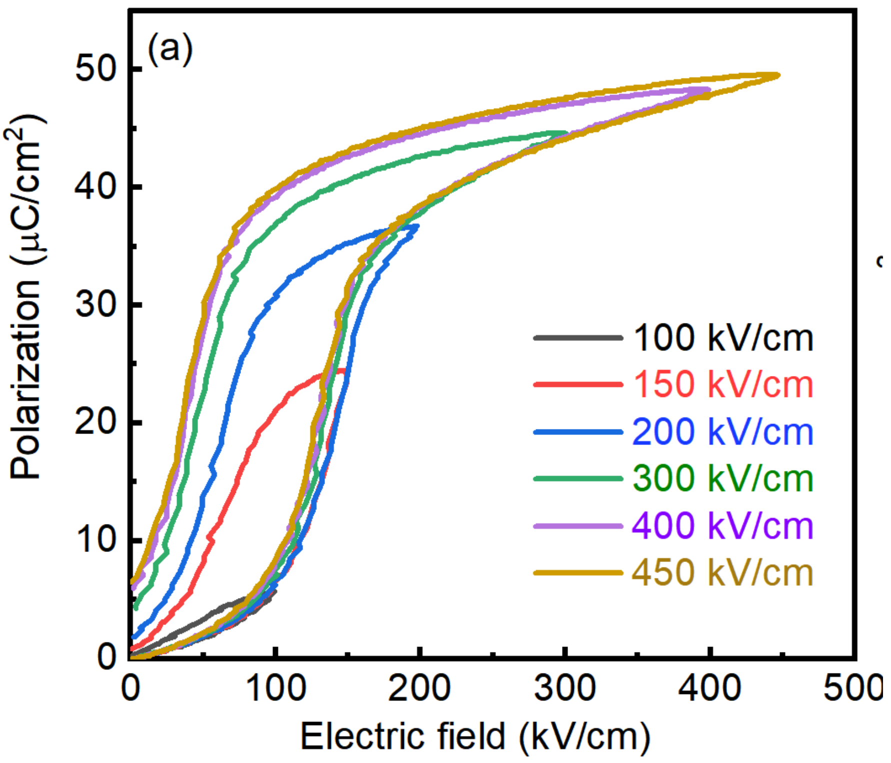

Fig.S6 Unipolar P-E loop for pure AN MLCCs measured at different electric fields. An obvious AFE characteristic is found in AN system. Besides, a large hysteresis is also detected in pure AN system, which is to the disadvantage of the improvement of energy storage and efficiency.

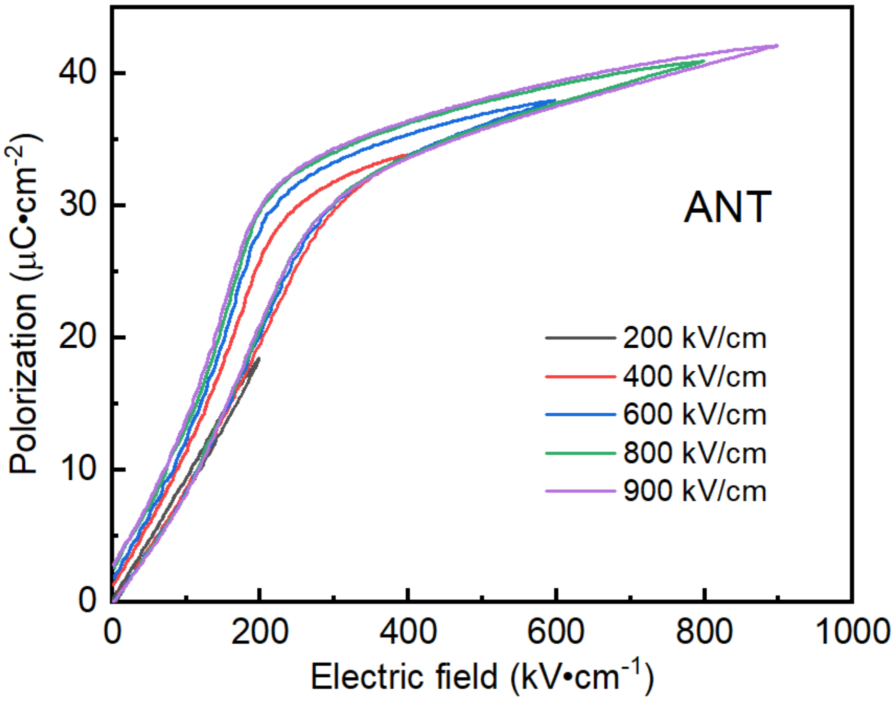

Fig.S7 Unipolar P-E loop for ANT MLCCs measured at different electric fields. Similar with AN sample, ANT system also shows an obviously AFE P-E loops. However, different from the AN system, the hysteresis of ANT system significantly reduces, and the breakdown strength is increase.

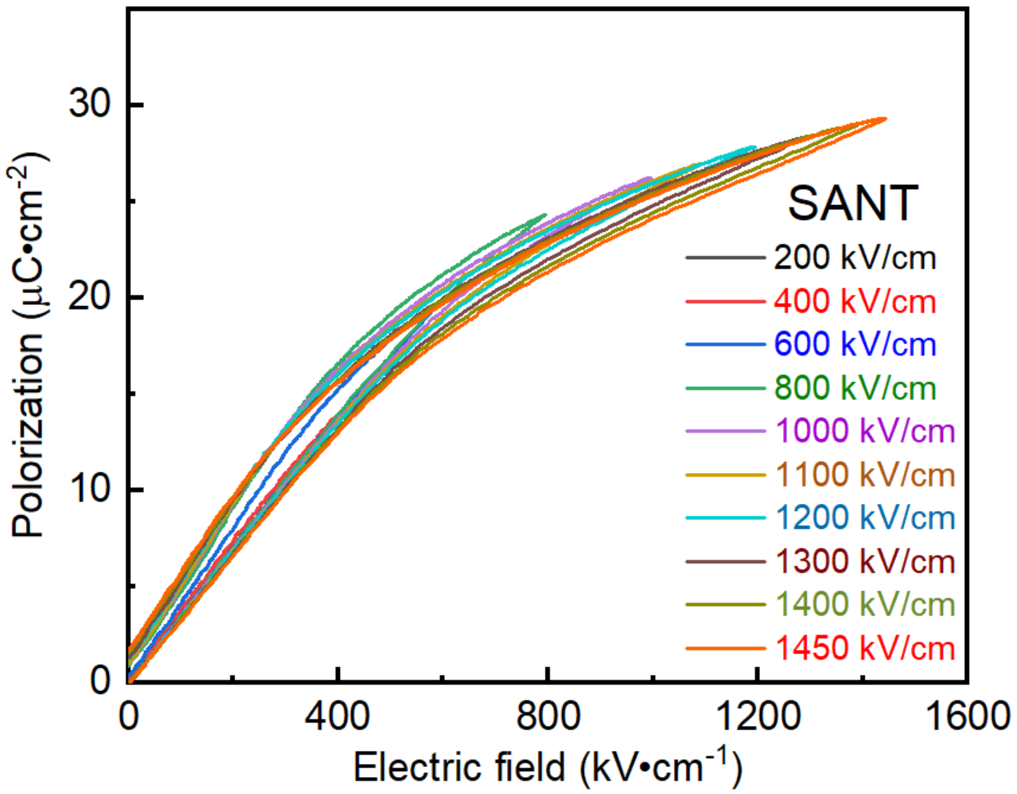

Fig.S8 Unipolar P-E loops for the samples of SANT MLCCs measured at different electric fields. A slim P-E loops was detected, indicating that SANT MLCCs possess a high efficiency, which is higher than that of AN and ANT systems. Besides, the breakdown strength is also far higher than that of AN and ANT systems, suggesting that SANT MLCCs possess an excellent energy storage density.

References

[1] Luo, N., Han, K., Cabral, M.J., Liao, X., Zhang, S., Liao, C., Zhang, G., Chen, X., Feng, Q., Li, J.F. & Wei Y. Constructing phase boundary in $AgNbO_3$ antiferroelectrics: pathway simultaneously achieving high energy density and efficiency. Nat. Commun. 11, 4824 (2020).

[2] Zhao, L., Liu, Q., Gao, J., Zhang, S. & Li, J.F. Lead-free antiferroelectric silver niobate tantalate with high energy storage performance. Adv. Mater. 29(31), 1701824 (2017).

[3] Li, G., Liu, H., Zhao, L., Gao, J., Liang, S., Li, J. & Zhu, J. Antiferroelectric order and Ta-doped $AgNbO_3$ with higher energy storage density. J. Appl. Phys. 125, 204103 (2019)

[4] Li, G., Liu, H., Zhao, L., Gao, J., Li, J., Yu, R. & Zhu, J. Atomic-scale structure characteristics of antiferroelectric silver niobate. Appl. Phys. Lett. 113, 242901 (2018).

[5] Park, S.E., Pan, M.J., Markowski, K., Yoshikawa, S. & Cros L.E. Electric field induced phase transition of antiferroelectric lead lanthanum zirconate titanate stannate ceramics. J. Appl. Phys. 82, 1798 (1997).

[6] Ji, Y., Li, Q., Zhuo, F., Yan, Q., Zhang, Y. & Chu X. Reversible and high-temperature-stabilized strain in $(Pb,La)(Zr,Sn,Ti)O_3$ antiferroelectric ceramics. ACS Appl. Mater. Interfaces. 11(35), 32135–32143 (2019).

[7] Liu, L., Shi, D., Knapp, M., Ehrenberg, H., Fang, L. & Chen J. Large strain response based on relaxor-antiferroelectric coherence in $Bi_{0.5}Na_{0.5}TiO_3$-$SrTiO_3$-$(K_{0.5}Na_{0.5})NbO_3$ solid solutions. J. Appl. Phys. 116, 184104 (2014).
